# Cadmium Ions’ Trace-Level Detection Using a Portable Fiber Optic—Surface Plasmon Resonance Sensor

**DOI:** 10.3390/bios12080573

**Published:** 2022-07-27

**Authors:** Bianca-Georgiana Şolomonea, Luiza-Izabela Jinga, Vlad-Andrei Antohe, Gabriel Socol, Iulia Antohe

**Affiliations:** 1 National Institute for Laser, Plasma and Radiation Physics (INFLPR), Atomiştilor Street 409, 077125 Măgurele, Ilfov, Romania; biancasolomonea@yahoo.com (B.-G.Ş.); izabela.jinga@inflpr.ro (L.-I.J.); 2 Faculty of Physics, Research and Development Center for Materials and Electronic & Optoelectronic Devices (MDEO), University of Bucharest, Atomiştilor Street 405, 077125 Măgurele, Ilfov, Romania; vlad.antohe@fizica.unibuc.ro; 3 Institute of Condensed Matter and Nanosciences (IMCN), Université catholique de Louvain (UCLouvain), Place Croix du Sud 1, B-1348 Louvain-la-Neuve, Belgium; 4 Academy of Romanian Scientists (AOSR), Splaiul Independenţei 54, 050094 Bucharest, Romania

**Keywords:** fiber optic—surface plasmon resonance (FO-SPR) sensors, polyaniline (PANI), bovine serum albumin (BSA), chitosan, cadmium detection

## Abstract

Environmental pollution with cadmium (Cd) is a major concern worldwide, with prolonged exposure to this toxic heavy metal causing serious health problems, such as kidney damage, cancer, or cardiovascular diseases, only to mention a few. Herein, a gold-coated reflection-type fiber optic–-surface plasmon resonance (Au-coated FO-SPR) sensor is manufactured and functionalized with (i) bovine serum albumin (BSA), (ii) chitosan, and (iii) polyaniline (PANI), respectively, for the sensitive detection of cadmium ions (Cd${}^{2+}$) in water. Then, the three sensor functionalization strategies are evaluated and compared one at a time. Out of these strategies, the BSA-functionalized FO-SPR sensor is found to be highly sensitive, exhibiting a limit of detection (LOD) for Cd${}^{2+}$ detection at nM level. Moreover, the presence of Cd${}^{2+}$ on the FO-SPR sensor surface was confirmed by the X-ray photoelectron spectroscopy (XPS) technique and also quantified consecutively for all the above-mentioned functionalization strategies. Hence, the BSA-functionalized FO-SPR sensor is sensitive, provides a rapid detection time, and is cheap and portable, with potential applicability for monitoring trace-level amounts of Cd within environmental or potable water.

## 1. Introduction

Contamination of rivers and seas with pesticides, plastics, drugs, or toxic heavy metals has reached worrying levels, mainly due to the rapid technology evolution and industrialization, as well as due to the waste resulting from agricultural facilities [1]. Heavy metals (i.e., cadmium, arsenic, lead, mercury, copper, chromium, etc.) are classified as environmental pollutants due to their high toxicity [2]. Heavy metal ions can seriously affect both humans and animals’ health, as well as producing damage to the environment. In particular, lead (Pb), chromium (Cr), mercury (Hg), and cadmium (Cd) are recognized as among the most dangerous water contaminants [3,4], especially because they are not biodegradable, thus posing a permanent threat to the medium [5]. Cadmium ions (Cd${}^{2+}$) can cause damage to the kidneys, liver, or heart, but they can also cause high blood pressure, cancer, or anemia. The main sources of Cd${}^{2+}$ found in nature come from fuel, batteries and chemical industries, or wastewater from coal [6,7]. Annually, 30,000 tons of Cd are released into the ecosystem, and 13,000 of them are produced by human activity. The World Health Organization (WHO) has set a safe limit of Cd${}^{2+}$ in both wastewater and soils for agriculture of 0.003 ppm or 0.003 mg/L [8].

The scientific community has shown a higher level of concern and implication regarding pollution with heavy metal ions in recent years. Therefore, several sensors designed for the quantitative and qualitative detection of heavy metal ions have been manufactured. The traditional heavy metal ion detection techniques are the spectrometric methods (i.e., graphite furnace atomic absorption spectrometry, flame atomic absorption spectrometry, inductively coupled plasma mass spectrometry, or atomic fluorescence spectrometry) and the chromatographic methods (i.e., gas chromatography, or high-performance liquid chromatography). Although these methods are stable and sensitive, they require complex and expensive instruments, costly maintenance, and highly qualified personnel to handle the equipment [9,10]. Alternatively, other analytical techniques have been reported to determine the amounts of Cd${}^{2+}$ in water samples, including electrochemical sensors [11,12,13,14], or sensors based on colorimetric and fluorescent detection [15,16]. These techniques can be integrated into a portable system that monitors real-time environmental pollutants, they are low-cost and sensitive, but they are not very stable to temperature and pH fluctuations [17].

Distinct to the detection techniques mentioned above, fiber optic (FO) technology based on the surface plasmon resonance (SPR) phenomenon is relatively new and offers many advantages, such as simplicity, portability, viability, low cost, and possibility for miniaturization [18,19,20]. Compared with the commercially available prism-based planar SPR devices (i.e., Biacore or Sensia), which are typically large and expensive laboratory-based instruments requiring well-trained personnel for their use [21], the FO-SPR technology is more amiable to miniaturization and user-friendliness, typically relying on cost-effective components. Lately, a compact and portable FO-SPR device (i.e., FOx Biosystems) has been released commercially, competing with the traditional SPR systems [22]. Surface plasmons (SPs) are electromagnetic waves that form along a plasmonic interface between a thin noble metal film (i.e., gold—Au) and a dielectric medium (i.e., solution to be analyzed) [23]. The devices based on the SPR phenomenon are therefore optical instruments that allow the detection and real-time monitoring of biomolecular interactions. The Au-coated FO is used to transport the light to the generated plasmonic interface. Any event that occurs at this interface (i.e., the interaction between a receptor and its specific target molecule) will trigger a change in the SPR signal, which can be further processed in a graph [23]. This is the reason that FO-SPR sensors have been widely used in medical diagnostic and environmental monitoring applications, in order to study molecular interactions and their binding specificity [24,25,26,27]. In the last few years, the FO-SPR sensors have been used for the detection of heavy metal ions in contaminated water [28,29,30]. However, there are only limited studies demonstrating the employment of FO-SPR systems of any configuration for Cd${}^{2+}$ sensing. R. Verma et al. designed a silver-coated transmission-type FO-SPR sensor for the detection of heavy metal ions (including Cd${}^{2+}$) using an indium thin oxide (ITO) intermediate layer and pyrrole/chitosan composite sensitive coating [31]. The sensitivity of the pyrrole/chitosan/ITO/Ag-coated FO-SPR sensor for Cd${}^{2+}$ was 2.589 nm/nM, with a limit of detection (LOD) of 0.129 nM. Four years later, P. Q. Zhu et al. fabricated a Cd${}^{2+}$ sensor by using allylthiourea-coated FO based on differential-temperature self-compensated technology [32]. The sensor has shown an LOD for Cd${}^{2+}$ of around 490 nM. More recently, T. Li and W. Feng proposed an FO-SPR sensor for the detection of Cd${}^{2+}$ in water based on a polyvinyl alcohol/titanium dioxide (PVA/TiO${}_{2}$) composite sensing film [33]. The sensitivity of this Ag-PVA/TiO${}_{2}$-coated sensor was found to be 48.2 nm/μM in the concentration range of 0.1–1 μM and the minimum detected concentration was less than 0.003 ppm of the international standard.

In this work, we report results on the design of a reflection-type FO-SPR sensor with performance that competes with the “state-of-the-art” devices for the plasmonic-based detection of Cd${}^{2+}$ in water samples. Herein, a Au-coated FO-SPR sensor is manufactured and functionalized with Cd${}^{2+}$-sensitive molecules: (i) bovine serum albumin (BSA) protein, (ii) chitosan biopolymer, and (iii) polyaniline (PANI) electro-conductive polymer, respectively. Therefore, the three sensors’ surface functionalization strategies are evaluated and compared. Out of these, the BSA/Au-coated FO-SPR sensor is found to be highly sensitive, exhibiting a limit of detection (LOD) at nM level. Moreover, the presence of Cd${}^{2+}$ on the FO-SPR sensor surface was confirmed by the X-ray photoelectron spectroscopy (XPS) technique and it was also quantified consecutively for all the above-mentioned functionalization strategies. The BSA-functionalized FO-SPR sensor is sensitive, it provides a rapid analysis time (within a few minutes), and it is cheaper and more portable compared to the classical SPR devices, being thus able to detect trace levels of Cd${}^{2+}$ in environmental or drinking water.

## 2. Materials and Methods

### 2.1. Reagents and Materials

All the reagents used in this work were of high purity and suitable for analytical applications. During the experiments, ultra-pure deionized water (DIW), purified by the TKA Mili-Q 50 system, was regularly employed. In order to fabricate the optical fiber (FO) sensors, a TEQS multimode FO of 400 μm diameter was acquired from Thorlabs. Acetone, aniline (C${}_{6}$H${}_{5}$NH${}_{2}$), ammonium persulfate ((NH${}_{4}$)${}_{2}$S${}_{2}$O${}_{8}$), toluene, hydrochloric acid (HCl), chitosan, bovine serum albumin (BSA), phosphate-buffered saline (PBS), acetic acid (CH${}_{3}$COOH), zinc chloride (ZnCl${}_{2}$), cobalt chloride (CoCl${}_{2}$), and cadmium iodide (CdI${}_{2}$) were supplied by Merck.

### 2.2. FO-SPR Portable System

**Figure 1:**
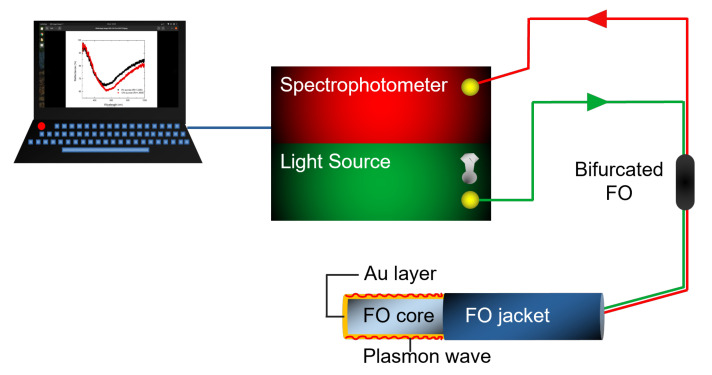
Schematic diagram of the portable FO-SPR sensing system.

The FO-SPR portable sensing system is schematically represented in Figure 1 and it is already well described in our previous work [25,26]. Briefly, an UV–VIS (Ultraviolet–Visible) spectrophotometer (AvaSpec ULS2048, Avantes, Apeldoorn, The Netherlands), a tungsten halogen light source (AvaLight, Avantes, Apeldoorn, The Netherlands), a bifurcated FO (Avantes, Apeldoorn, The Netherlands), as well as an interchangeable FO-SPR sensor, are included in the system. The spectrophotometer has a wavelength range of 200–1100 nm, a sensitivity of 310,000 counts/μW per ms integration time, a signal/noise ratio of 200:1, and an integration time of 1.05 ms to 10 min. This spectrophotometer is further connected to a laptop and used to measure the light reflected by the FO sensing tip. If there are any changes occurring at the gold (Au) surface, there will be a shift in the SPR response. The FO-SPR sensor’s fabrication protocol was also previously described [18,25], consisting of a few steps. In short, after cutting the FO into small pieces, SPR-sensitive zones of 0.6 cm are created and uncladded in acetone. Then, the FO substrates are Au-coated using a magnetron sputtering device (Quorum Q150R ES, East Sussex, UK) equipped with a quartz crystal oscillator (QCM) for real-time film thickness monitoring. Finally, the as-prepared Au-coated FO sensors are used for the implementation of the three Cd${}^{2+}$ detection strategies.

### 2.3. Sensor Functionalization Strategies for Cd${}^{2+}$ Detection

Strategy I: The Au-coated FO-SPR sensors were immersed in 5 μM BSA protein in PBS buffer (pH 7.4) for 1 h. Due to the physisorption of BSA on the Au surface, the BSA will form a coating on the Au-coated FO substrate. The interaction of BSA with Cd is well known; the latter is covalently binding to the amino acid protein constituents [34].

Strategy II: The Au-coated FO-SPR sensors were immersed for 1 h in 5 μM chitosan dissolved in 2% acetic acid in DIW. This protocol was inspired by the literature [31] and adapted to the FO geometry. Chitosan is a good sorbent for heavy metal ions, including Cd${}^{2+}$ [35].

Strategy III: Polyaniline (PANI) films were synthesized on the Au-coated FO-SPR substrates using a chemical oxidative polymerization method [36]. The Au-coated FO were immersed in an aqueous solution of 1 M (NH${}_{4}$)${}_{2}$S${}_{2}$O${}_{8}$ and 1 M HCl, and afterwards a solution of 0.1 M aniline in toluene was poured over the previously prepared mixture. The sensors were kept immersed in the solution for 10 min at a constant temperature of 25 ${}^{\circ }$C. A green film was observed after the immersion time, which is proof of the formation of the Emeraldine state of PANI on the Au-coated FO. The sensors were taken out after the mentioned immersion time and were washed in DIW and then dried. PANI is also a good adsorbent, currently used for removing various types of heavy metal ions and dyes from aqueous solutions [37].

Cd${}^{2+}$ dilutions: Firstly, we prepared an aqueous 1 mM CdI${}_{2}$ stock solution. Further, all the heavy metal ion concentrations were independently prepared by diluting the stock solution. The refractive index (RI) values of all solutions were verified by an Abbe refractometer having a resolution of 0.001, and it was found that they all had a similar RI to the DIW (i.e., 1.333). Next, the functionalized FO-SPR sensors were used for the detection of various concentrations of Cd${}^{2+}$ ions (i.e., 0, 0.06, 0.12, 0.25, 0.5, and 1 μM) in DIW. The functionalized FO-SPR sensor was kept for 5 min in each Cd${}^{2+}$ dilution. Every Cd${}^{2+}$ concentration was measured three times independently using the freshly prepared sensors. The functionalized FO-SPR’s sensitivity for Cd${}^{2+}$ was evaluated by plotting the SPR wavelength shift as a function of the Cd${}^{2+}$ concentration, followed by linearly fitting the obtained calibration curve. To calculate the limit of detection (LOD), we employed the 3σ/S formula, where σ and S are the standard deviation of the lowest concentration measured and the slope of the linear fit, respectively [25,38].

Zn${}^{2+}$ and Co${}^{2+}$ dilutions: ZnCl${}_{2}$ and CoCl${}_{2}$ aqueous solutions were prepared with a concentration of 1 μM to evaluate the selectivity of the FO-SPR sensor.

### 2.4. Characterization of the FO-SPR Surfaces

The FO-SPR sensor’s surfaces were investigated by means of X-ray photoelectron spectroscopy (XPS), utilizing Thermo Fisher Scientific ESCALAB Xi${}^{+}$ equipment featuring a multichannel hemispherical electron analyzer (dual X-ray source) working with Al Kα radiation (1486.2 eV). Binding energies were calculated with reference to the C-(C,H) component of the C 1s peak set at 284.8 eV. XPS spectra were interpreted using the NIST Database, as well as the Handbook of XPS [39]. Prior to the XPS analysis, the FO-SPR sensors were outgassed in vacuum (at a pressure lower than 2 × 10${}^{-6}$ Pa) in the pre-chamber of the XPS setup at room temperature to eventually remove the chemisorbed water from the sensor’s surfaces. Notably, the XPS measurements were carried out on all FO-SPR sensors after Cd${}^{2+}$ detection, in order to quantitatively evaluate the amount of Cd absorbed by the FO-SPR sensors during the Cd${}^{2+}$ sensing measurements.

## 3. Results and Discussion

### 3.1. Cd${}^{2+}$ Detection Strategies: Sensitivity and Limit of Detection Evaluation

As mentioned in Section 2.3, three surface functionalization strategies were applied and the as-prepared Au-coated FO-SPR sensors were employed for detecting various concentrations of Cd${}^{2+}$ (0, 0.06, 0.12, 0.25, 0.5, and 1 μM) in DIW.

**Figure 2:**
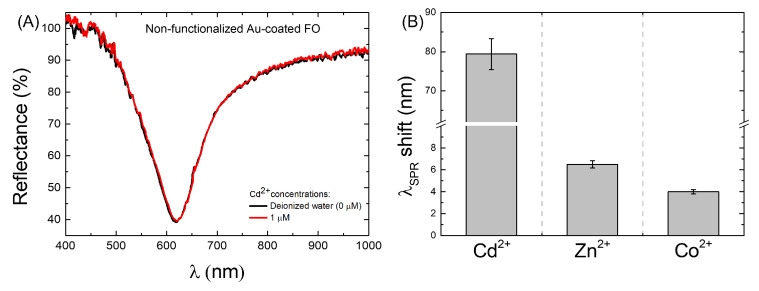
FO-SPR sensor specificity measurements. (**A**) Control test, showing that, in the absence of any Cd${}^{2+}$-sensitive layer on the Au-coated sensor, only an SPR wavelength shift of ∼2 nm is recorded for the 1 μM Cd${}^{2+}$ solution. (**B**) Evaluation of the BSA/Au-coated FO-SPR sensor’s selectivity for detection of 1 μM Cd${}^{2+}$, Zn${}^{2+}$, and Co${}^{2+}$ metal ions, respectively, in aqueous solutions. The error bars represent standard deviation (*n* = 3).

The sensors’ specificity was first evaluated by using a non-functionalized Au-coated FO-SPR sensor to detect the highest Cd${}^{2+}$ concentration (1 μM). Figure 2A shows the SPR spectral dips obtained with the non-functionalized Au-coated FO-SPR sensor in DIW (black curve) and 1 μM Cd${}^{2+}$ concentration (red curve). It was found that the two SPR spectral dips almost overlapped, with a tiny wavelength shift of only 2 nm observed, proving the Cd non-specificity on the Au-coated FO-SPR sensor. However, the observed negligible variation in the SPR wavelength for the non-functionalized sensor may have been due to the presence of very few Cd${}^{2+}$ ions on the Au-coated sensing area.

**Figure 3:**
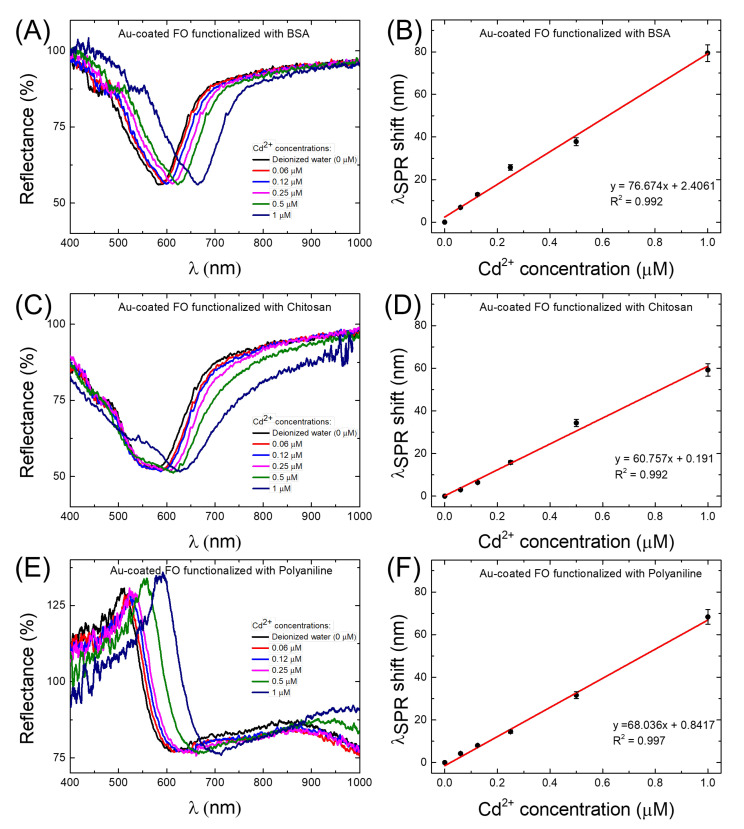
SPR spectra for varying concentrations from 0 to 1 μM of Cd${}^{2+}$ using (**A**) BSA, (**C**) chitosan, and (**E**) PANI/Au-coated FO-SPR sensors. (**B**,**D**,**F**) Corresponding calibration curves of the FO-SPR sensors. The error bars represent standard deviation (*n* = 3).

Figure 3 presents the SPR spectral dips and corresponding calibration curves of the FO-SPR sensors coated by BSA/Au (Figure 3A,B), chitosan/Au (Figure 3C,D), and PANI/Au (Figure 3E,F), respectively. Notably, a clear wavelength right-shift of the SPR dip with increasing Cd${}^{2+}$ concentration could be observed for the BSA/Au-coated FO-SPR sensor (Figure 3A). The obtained calibration curve in this case (Figure 3B) revealed the sensor’s sensitivity (the slope of the linear regression) of 76.67 nm/μM (calculated with a coefficient of determination R${}^{2}=$ 0.992), with an estimated LOD of 7.1 nM. In contrast, the chitosan/Au-coated FO-SPR sensors exhibited a wavelength right-shift in the SPR spectral dips in a narrower range compared to the BSA/Au-coated sensor (Figure 3C), leading to a lower sensitivity of 60.75 nm/μM (R${}^{2}=$ 0.992) and an LOD of 9.4 nM (Figure 3D). Ultimately, for the PANI/Au-coated FO-SPR sensor, a wavelength right-shift in the SPR dip of approximately 68 nm was observed for the highest Cd${}^{2+}$ concentration of 1 μM (Figure 3E). In the latter scenario, the sensitivity was estimated to be 68.03 nm/μM (R${}^{2}=$ 0.997), with an LOD of 8.8 nM (Figure 3F). The obtained data are also summarized in Table 1.

As can be also noticed from Figure 3A,C,E, the overall shape and broadness of the SPR spectral dips was different among the three tested FO-SPR sensor functionalization strategies (i.e., BSA, chitosan, and PANI). This observation can be mainly attributed to the variations in the molecular weight of the functional layers, from ∼66 kDa for BSA to more than 170 kDa for PANI polymers [40,41,42], reported to generally influence the SPR reflectivity and dip flatness [21]. Moreover, the more flattened dips observed for the PANI/Au-functionalized FO-SPR sensors can be explained by the high RI value of PANI film, which contributes to the SPR dips broadening [31,43,44].

Based on the observed behavior, it can be clearly noticed that all the prepared sensors are capable of detecting nM levels of Cd${}^{2+}$ in DIW. In all the tested configurations, the main sensing mechanism relies on either physical absorption or chemisorption of Cd${}^{2+}$ by the functional layer, as similarly reported elsewhere [28,34,37]. Consequently, the absorbed heavy metal ions on the sensor’s surface trigger changes in the optical properties of the functional layer, modifying further the RI value close to the plasmonic interface and thus causing shifts in the SPR spectral dips, as observed in Figure 3. However, it is well known that the covalent binding of the Cd${}^{2+}$ to the amino acid protein constituents of the BSA is more powerful [34] than the *RedOx* protonation reactions induced by the presence of Cd${}^{2+}$ ions, typically associated with the chitosan and PANI polymers, respectively [25]. Hence, the improved absorption of the Cd${}^{2+}$ ions on the FO surface might explain the better performance of the BSA/Au-coated FO-SPR sensor in terms of sensitivity and LOD. Additionally, Zn${}^{2+}$ and Co${}^{2+}$ aqueous solutions with a concentration of 1 μM were used to evaluate the selectivity of the FO-SPR sensor functionalized with BSA protein. The SPR wavelength shift of the sensor to 1 μM Cd${}^{2+}$ (∼80 nm) is much greater than those of 1 μM Zn${}^{2+}$ (∼6.5 nm) and of 1 μM Co${}^{2+}$ (∼4 nm), respectively, as can be seen in Figure 2B, indicating that the sensor has excellent selectivity to Cd${}^{2+}$.

### 3.2. Characterization of the FO-SPR Surfaces

**Figure 4:**
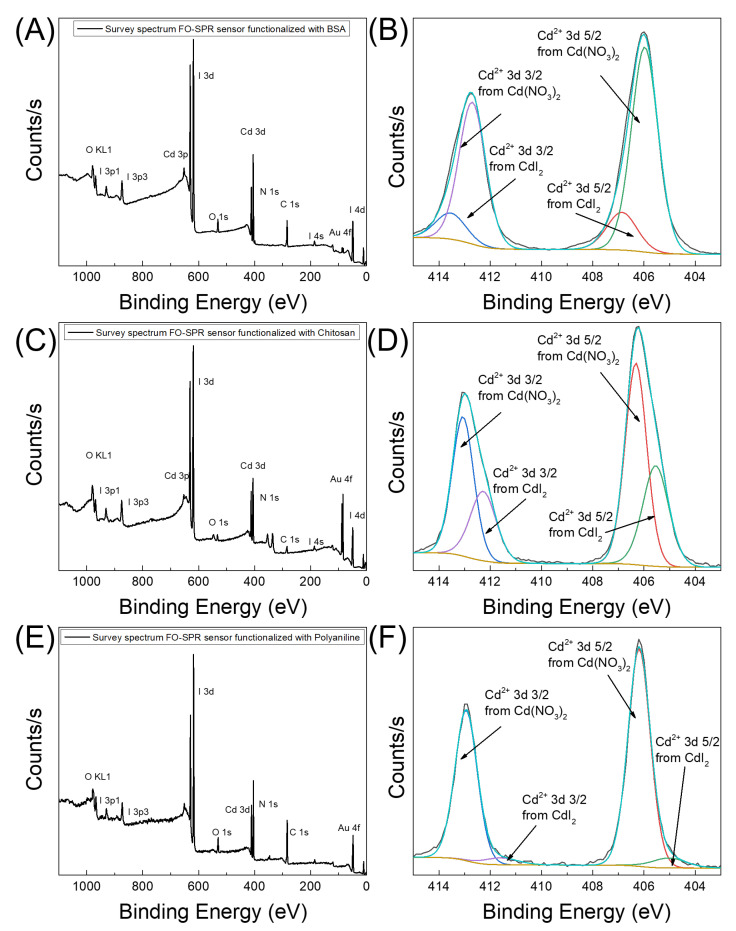
XPS spectra of the FO-SPR sensors’ surfaces. Wide scan of (**A**) BSA/Au, (**C**) chitosan/Au, and (**E**) PANI/Au-coated FO-SPR sensors after 1 μM Cd${}^{2+}$ detection, respectively; (**B**,**D**,**F**) Corresponding core-level spectra of the Cd${}^{2+}$ immobilized on the functionalized Au-coated FO-SPR sensors.

To evaluate the amount of absorbed Cd${}^{2+}$ species on the surface of the Au-coated FO-SPR sensors functionalized with BSA, chitosan, and PANI, the surface chemistry of the sensors was elementally and quantitatively analyzed by XPS. The XPS spectra of the tested FO-SPR configurations, after detecting 1 μM of Cd${}^{2+}$, are presented in Figure 4. The atomic percentage of Cd${}^{2+}$ was calculated by dividing the photoelectron peak intensity for each element by the corresponding Relative Sensitivity Factor (RSF) [45].

Figure 4A,C,E show the wide scan plots of the Au-coated FO-SPR sensors functionalized with BSA, chitosan, and PANI, respectively. As can be noticed, carbon (C 1s, 284.8 eV) and nitrogen (N 1s, 398 eV) signals are consistently presented in all cases, being attributed to the existence of the organic compounds on the Au-coated FO-SPR sensors’ surfaces (i.e., BSA, chitosan, and PANI), while the oxygen (O 1s, 532 eV) signal can be assigned to an associated oxidative state of the functional layers. Additionally, the wide scans reveal also Au peaks emanating from the Au plasmonic thin film deposited on the FO-SPR sensors’ surfaces, as well as iodine (I) and cadmium (Cd) signals obviously attributed to the presence of these species on the sensors’ surfaces. To quantify the absorbed Cd${}^{2+}$ on the FO-SPR sensors’ surfaces, Figure 4B,D,F display the corresponding Cd${}^{2+}$ core-level spectra. From the latter plots, the detected percentage of Cd${}^{2+}$ on the sensors’ surfaces was calculated to be 21% (Figure 4B), 14.7% (Figure 4D), and 16% (Figure 4F), for the BSA/Au, chitosan/Au, and PANI/Au-coated FO-SPR sensors, respectively.

The obtained data are collected in Table 1. Notably, a clearly increasing tendency of the Cd${}^{2+}$ atomic percentage with increasing sensitivity values among the tested FO-SPR configurations can be noticed. The observed results are in agreement with our previous conclusions, demonstrating that the BSA/Au-coated FO-SPR sensor has a high affinity for Cd${}^{2+}$ absorption, compared with the other two sensors, resulting in better sensitivity and subsequently LOD values (see Table 1). Based on the results obtained, we can conclude that the BSA/Au-coated FO-SPR sensor is the most sensitive configuration capable of detecting trace-level amounts of Cd${}^{2+}$ in DIW.

The sensitivity value of 76.67 nm/μM, obtained with the BSA-functionalized sensor, competes with other state-of-the-art FO-SPR sensor configurations employed so far for Cd${}^{2+}$ detection in water samples, as indicated in Table 2. However, a notably better sensitivity value was obtained by Li et al., albeit only in an extremely narrow concentration range (i.e., 0–40 nM), explained by more efficient Cd${}^{2+}$ absorption on the sensor surface for this concentration range, assured by the large specific surface area given by the PVA/TiO${}_{2}$ sensing film [33]. Moreover, Verma et al. obtained encouraging results for Cd${}^{2+}$ detection while employing a transmission-type FO-SPR configuration in conjunction with a hybrid polymer/bio-polymer (i.e., polypyrrole/chitosan) functional bilayer, in comparison to a relatively more complex and expensive FO-SPR setup [31]. Meanwhile, the reflection-type FO-SPR sensor proposed in this work has a number of features that make it significant, i.e., its fabrication is very simple, it is low-cost, it works in the visible region of the electromagnetic spectrum, it can be used in out-of-the-lab on-site applications, and it is also quite sensitive for Cd${}^{2+}$ detection.

## 4. Conclusions

In this work, we propose a Au-coated reflection-type FO-SPR sensor functionalized with (i) BSA, (ii) chitosan, and (iii) PANI to determine the amount of Cd${}^{2+}$ in DIW. Among all the applied strategies, the BSA/Au-coated FO-SPR sensor showed promising results, detecting Cd${}^{2+}$ with a sensitivity of 76.67 nm/μM and an LOD of 7.1 nM. This sensor could be successfully used outside the laboratory facility for on-site environmental monitoring and drinking water quality control.

## Figures and Tables

**Table 1:** Performance comparison between the three differently prepared FO-SPR sensors.

FO-SPR Sensor Type	Sensitivity	Limit of Detection (LOD)	Atomic Percentage of
	[nm/μM]	[nM]	Absorbed Cd${}^{2+}$ [%]
BSA/Au	76.67	7.1	21
PANI/Au	68.03	8.8	16
Chitosan/Au	60.75	9.4	14.7

**Table 2:** Performance comparison of different FO-SPR sensors functionalized with layers sensitive to Cd${}^{2+}$.

FO-SPR Sensor Configuration	Cd${}^{2+}$ Sensitive Layers	Sensitivity	Concentration
		[nM/μM]	Range [μM]
Reflection [this work]	BSA/Au	76.67	0–1
Reflection [33]	Ag-PVA/TiO${}_{2}$	48.2	0–1
		315.2	0–0.04
Transmission [46]	SnO${}_{2}$-MoS${}_{2}$	0.03	0–100
Transmission [47]	SnO${}_{2}$/Ag	23.71	0–10
Transmission [31]	Pyrrole/chitosan/ITO/Ag	146.8	0–1.8

## Data Availability

The data used and/or analyzed during the current study are available from the corresponding authors on reasonable request.

## Abbreviations

The following abbreviations are used in this manuscript:

INFLPR	National Institute for Laser, Plasma and Radiation Physics
R&D	Research & Development
MDEO	R&D Center for Materials and Electronic & Optoelectronic Devices
IMCN	Institute of Condensed Matter and Nanosciences
UCLouvain	Université catholique de Louvain
WHO	World Health Organization
SPs	Surface Plasmons
SPR	Surface Plasmon Resonance
FO-SPR	Fiber Optic–Surface Plasmon Resonance
RI	Refractive Index
ITO	Indium Tin Oxide
LOD	Limit of Detection
PVA	Polyvinyl Alcohol
BSA	Bovine Serum Albumin
PANI	Polyaniline
UV–VIS	Ultraviolet–Visible
QCM	Quartz Crystal Microbalance
RSF	Relative Sensitivity Factor
DIW	Deionized Water
SEM	Scanning Electron Microscope
XPS	X-ray Photoelectron Spectroscopy
AOSR	Academy of Romanian Scientists
UEFISCDI	Executive Unit for Financing Higher Education, Research, Development and Innovation
